# Cilia in autophagy and cancer

**DOI:** 10.1186/s13630-016-0027-3

**Published:** 2016-02-03

**Authors:** Muqing Cao, Qing Zhong

**Affiliations:** Center for Autophagy Research; Department of Internal Medicine, University of Texas Southwestern Medical Center, Dallas, TX USA

**Keywords:** Cilia, Cell cycle, Cilia-related signaling, Autophagy, Cancer

## Abstract

Cancer cells are distinguished from normal cells by increased proliferation and metabolism, loss of polarity control, and the potential to invade other tissues of the body. As hubs of signaling transduction, primary cilia have been linked to diverse developmental and degenerative disorders. Interestingly, loss of cilia has been observed in multiple malignant tumors, suggesting a potential suppressive role of cilia in cancer development. More recently, emerging studies began to unveil the bidirectional interaction of cilia and autophagy, a basic cellular clearance and recycling mechanism to regulate cell homeostasis. Here, we summarize the interplay between cilia and autophagy and discuss the roles of cilia in both autophagy and cancer.

## Background

Cilia/flagella are eukaryotic cell organelles protruding from cell surface into environment. Most vertebrate cells assemble a single primary cilium, when they exit from the cell cycle into differentiated or quiescent status [1]. This ~5 μm tall, microtubule-based protrusion is essential for multiple signaling transductions [2, 3]. Autophagy is a destructive cellular process to degrade disordered cell organelles and protein aggregates, and maintain cellular homeostasis. More recently, cilia are attracting interests as structures having bidirectional interaction with autophagy. This review will discuss the relationship between cilia and autophagy and emphasize the function of cilia in cancer development.

### The connections between cilia and cancer

**Fig. 1:**
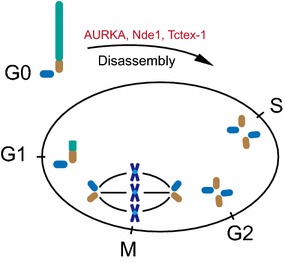
The centriole–cilium cycle in mitosis. Primary cilium is assembled on the distal end of mother centriole during G0/G1 phase. Before S-phase entry, Aurora A, Nde1, and Tctex-1 trigger the disassembly of primary cilium. Delayed S-phase re-entry is observed in the cells that have defects in cilia disassembly

Primary cilium possesses an axoneme consisting of nine doublet microtubules, which is surrounded by a specialized membrane [4]. In interphase, cilium biogenesis is initiated by the attachment of a Golgi-derived membrane vesicle on the distal end of mother centriole [5, 6]. Subsequently, the nucleated axoneme buds from mother centriole and bends the cell membrane to form the structure [5, 6]. Considering that centrosomes direct spindle formation in mitosis, cilia must be disassembled before mitosis to liberate the captive centriole and to promote the formation of spindle [7–10]. The presence of cilia can suppress abnormal cell growth by restricting cell cycle (Fig. 1). Although the ciliary membrane is continuous with cell membrane, the lipid and protein compositions of ciliary membrane are different from cell membrane compositions [11–15]. The specialized ciliary membrane makes cilia capable of transducing multiple cellular signaling [2, 16–21].

Since primary cilia have the ability to influence cell cycle and modulate cilia-related signaling transduction, dysfunction of cilia has long been proposed as a prerequisite step of cancer development [7, 22]. In contrast to normal cells, cilia are lost in multiple cancer types [22]. Clinical data also show that cilia formation is compromised in multiple human cancers including breast cancer, cholangiocarcinoma, melanoma, pancreatic cancer, prostate cancer, and renal cell carcinoma [17, 22–30]. These observations suggest that cilia play a suppressive role in cancer development. Although defective cilia and cancer are always associated, a direct role of cilia in tumorigenesis is still elusive.

The negative correlation between cilia and cell cycle has been discovered for many decades. The studies in Snell’s group provided the first molecular link between cilia disassembly and cell cycle progression [7, 31]. They found that the disassembly of *Chlamydomonas* cilia requires protein CALK, a member of Aurora kinase family, which promotes cell cycle [31]. In 2007, Golemis’s group optimized a system to study the mechanisms of cilia disassembly [32]. Briefly, cells were treated by serum starvation to induce cilia formation. Serum was added into the medium to trigger cilia disassembly and cell cycle re-entry. Like the observation in *Chlamydomonas*, they found that mammalian cells also shorten their cilia through Aurora kinase-dependent pathway. Upon serum stimulation, HEF1 activated Aurora A, which phosphorylated HDAC6 to promote cilia disassembly during cell cycle re-entry [32, 33]. According to these findings, a straightforward question will be whether cell cycle progression is blocked with defective cilia disassembly? Tsiokas and colleagues found that knockdown of *Nde1*, a mother centriole-localized protein, led to elongated cilia in mammalian cells [34]. Interestingly, G0 cells with longer cilia by depletion of Nde1 delayed cell cycle re-entry after serum addition. To confirm the delayed re-entry was caused by cilia, the group co-knockdown *Nde1* with either *Ift88* or *Ift20*, two essential genes for ciliogenesis, and found that the inhibitory effect was reversed [34]. Concomitantly, Sung’s group showed that Tctex-1 localizes to transition zone after phosphorylation at Thr94, where it promotes cilia disassembly before S-phase entry [35]. Consistently, depletion of Tctex-1 resulted in delayed cilia disassembly along with delayed cell cycle re-entry in ciliated cells, but not in non-ciliated cells [35]. Both of the studies indicate a suppressive role of cilia in cell cycle progression (Fig. 1), raising the possibility that loss of cilia promotes unrestricted cell cycle progression in cancer cells.

**Fig. 2:**
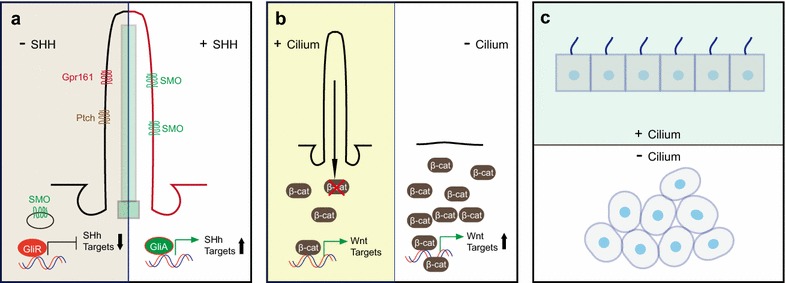
Cilia-related signaling pathways. **a** Several Shh proteins are located in cilia. In repression state, Gpr161 and Ptch are present in cilia, but SMO is excluded from cilia. Gli proteins are cleaved as the repressive form to inhibit Shh target gene transcription. With the binding of Shh ligand, GPR161 and Ptch move out from cilia, but SMO is transported into cilia. The stabilized Gli proteins are activated to trigger the transcription of Shh target genes. **b** Primary cilium provides an additional brake for canonical Wnt signaling by promoting the degradation of β-catenin. **c** Cilia are involved in the regulation of PCP signaling, disruption of which leads to abnormal cell orientation. Abnormal cell polarity is a major symptom of diseases with defective cilia

The other important function of primary cilium is its ability to regulate multiple signaling pathways, the dysfunctions of which are associated with a number of cancers [22]. As a cilia-dependent pathway (Fig. 2a), Sonic Hedgehog (Shh) signaling has important functions in guiding embryonic development by regulating cell differentiation and proliferation [20, 36–39]. In the absence of Shh ligand, membrane proteins Patched and Gpr161 are localized to cilia. On the other hand, the most majority of Smoothened is excluded from cilia, though a basal level of protein is thought to be traffic through cilia as well [37, 39]. Shh transcriptional factors Gli2 and Gli3 are cleaved to Gli2R and Gli3R repressor forms and inhibit Shh downstream transcriptions [40]. Upon the Shh ligand binding, Patched and Gpr161 are moved out from cilia, but Smoothened is accumulated in cilia. Consequently, the stabilized Gli2 and Gli3 can be activated as Gli2A and Gli3A, which trigger the transcription of Gli1 and other Shh target genes [39, 40]. In several types of cancers, the abnormal activations of Shh are observed [22, 41–43]. For instance, dysregulated activation of Shh contributes to basal cell carcinoma and medulloblastoma development [41, 43]. It is interesting that primary cilia are either positive or negative regulators of Shh-related oncogenesis, depending on the initiating oncogenic mutations [41, 43]. Wnt signaling is critical to animal development and homeostasis [44]. Upregulation of Wnt signaling has also been linked to tumorigenesis [22, 42, 44]. Although the functions of cilia in Wnt signaling are still controversial, it seems that both canonical and non-canonical Wnt signaling can be regulated by cilia (Fig. 2b, c). Down regulation or loss of ciliary proteins, including BBS1, BBS4, Kif3a, IFT88, and OFD1, leads to accumulation of β-catenin, which subsequently increases the transcription of Wnt target genes in *Zebrafish* embryos and mouse cells and embryos [21, 44, 45]. Interestingly, *Ift88*, *Ift172*, and *Kif3a* mutant mice, which also lack functional primary cilia, failed to show any phenotype caused by upregulated Wnt signaling [46]. Considering that Wnt signaling is strictly regulated in specific developmental stage and tissue, these results may still reflect the ability of cilia to regulate Wnt signaling. In contrast to canonical Wnt, non-canonical signaling is β-catenin independent and involved in the regulation of cell polarity [47–49]. Cystic diseases are well-established models of human diseases caused by dysfunctional cilia. Similar with tumor cells, a typical symptom of cystic organs is a loss of cell polarity [17, 50–52]. Consistent with these observations, planar cell polarity (PCP) mutant phenotypes, including open eyelids and disorganized stereocilia, are found in *Bbs1*, *Bbs4*, and *Bbs6* defective mice [53]. Loss of two cilia-related proteins, Ivn/NPHP2 and OFD1, also leads to PCP-regulated convergent extension defects in vertebrates [48, 54]. All of these studies demonstrated that cilia are capable of regulating both canonical and non-canonical Wnt signaling [18, 44, 46, 55–57].

Including Shh and Wnt, the list of cilia-related signalings is growing fast. TGF-beta, Hippo, and notch signaling are also connected with cilia [17, 58–61]. In addition to the function of receiving and transducing signaling, recent studies in *Chlamydomonas* showed cilia can also release signaling active vesicles and act as signaling transmitting organelles to regulate the behavior of other cells [62–64]. All these studies show a strong link between cilia and cancer development. However, if and how cilia function in tumorigenesis remains unclear. Tumors are highly heterogeneous tissues and consisted of different cell types, including tumors cells and tumor-associated fibroblasts, endothelial cells, and immune cells [65–69]. These cells and extra-cellular matrix constitute tumor stroma [66, 69, 70]. Functioning as sensing organelles, loss of cilia may alter the signaling network and cell–cell communications inside tumor stroma. Involved in multiple signaling, the function of cilia in tumorigenesis will be far more complex than it appears now and will not be limited to the cell cycle regulation and polarity control. If and how cilia contribute to cancer development remains an important question to be addressed.

### The bidirectional interplay between cilia and autophagy

**Fig. 3:**
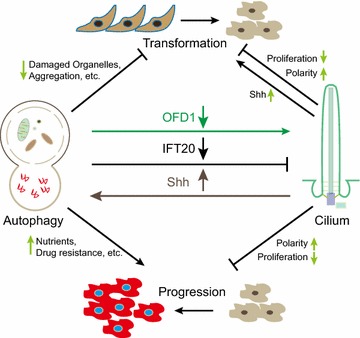
Illustrative model of the links between cilia, autophagy, and cancer. Autophagy has dual roles in ciliogenesis by selectively degrading cilia essential protein IFT20 to suppress cilia formation, or degrading suppressive protein OFD1 to promote cilia formation. Inversely, cilia can enhance autophagy through cilia-dependent Shh signaling. Both cilia and autophagy are proposed as regulators for cancer development. The cross-talk between cilia and autophagy may provide new applications for cancer drug discovery

Autophagy has essential functions in multiple physiological processes [71]. The relationship of cilia and autophagy has been missing for a long period. A set of recent studies established the relationship of cilia and autophagy (Fig. 3) [72–74]. In contrast to cells in vivo, most in vitro cultured cells do not express cilia. Early studies showed that serum withdrawal leads to cell cycle exit and induces ciliogenesis. Interestingly, serum starvation can also trigger autophagy. The most natural question to ask is if these two concurrent events are related? Tang and colleagues demonstrated that OFD1, a ciliopathy protein, was degraded by autophagy to promote cilia formation upon serum starvation [72, 75]. OFD1 is localized to two cilia-related subcellular structures, distal end of centrioles and centriolar satellites [72, 76–78]. The centriolar OFD1 is thought to maintain centriolar length and integrity, which is required for cilia formation [76]. However, the function of satellite pool was unclear. Tang showed that autophagy largely eliminated the satellite OFD1 but not centriolar OFD1. Inhibition of autophagy attenuated satellite OFD1 degradation and led to lower ciliogenesis rate and shorter cilia. Consistently, depletion of OFD1 by RNA interference dramatically increased cilia formation in murine embryonic cells and restored ciliogenesis in MCF7 cells, a breast cancer cell line originally lacking cilia [72]. All these data demonstrated a suppressive role of satellite OFD1 in cilia formation and suggested a positive role of autophagy in ciliogenesis. In contrast to stimulated autophagy, Cuervo’s group showed that basal level autophagy acts as a negative regulator for ciliogenesis by degrading IFT20, ciliary essential protein [74]. The switch of basal autophagy and stimulated autophagy may potentiate autophagy positively or negatively controlling cilia formation in response to environmental changes [79]. However, a surprising aspect in these two studies is that the ratio of ciliated cells to ciliary length in ATG5^−/−^ autophagy-defective MEF cells are quite different [72, 74]. Given that the confluence of cells has strong influence on cilia formation, one possible explanation of the observations might be attributed to the different status of cell density in these two studies. MTOR is a known negative regulator of autophagy [80]. Wang et al. showed that MTOR activity is upregulated in cilia-suppressed cells, also suggesting that lower autophagy level is associated with attenuated cilia formation [81]. Consistent with the observation, they also showed that upregulating autophagy activity prompted cilia elongation and downregulating autophagy activity led to shortened cilia in kidney cells [81]. Taken together; these studies demonstrated that autophagy can serve as a dual-role regulator of ciliogenesis by alternatively eliminating ciliary essential protein(s) or its suppressive protein(s) [75, 79]. Understanding of the mechanisms that controls the autophagy switch to turn on/off of cilia formation will be an important question for future studies.

Despite the function of autophagy in ciliogenesis, cilia and cilia-related Shh signaling are potential regulators of autophagy. Several components of autophagic machinery localize around ciliary or periciliary region [74, 79]. ATG16L, AMBRA1, LC3, GABARAP, and VPS15 staining showed discrete puncta along cilia [74, 79]. ATG14, VPS34, ATG7, and ATG5 are found at the basal body region [74, 79]. Regarding the vesicular activity of ciliary pocket, the presence of autophagic initiating molecules around cilia suggests ciliary area as a possible novel origin of autophagosome formation to activate autophagy. Consistently, lower autophagy activity is observed in IFT20 knockdown MEF cells and IFT88 knockout kidney epithelial cells, both of which have defects in ciliogenesis [74, 79]. Interestingly, the activation of Shh rescued the defective autophagy flux, indicating that cilia upregulate autophagy through cilia-dependent Shh signaling [74, 79]. In another study, cilia-suppressed cells also showed repressed autophagy, which might be resulted from enhanced MTOR activity [81]. In ciliated neuron and smooth muscle cells, autophagy activation was also observed after the upregulation of Shh by its ligand binding [82, 83]. One argument against the positive role of cilia in promoting autophagy comes from the studies of Pancreatic ductal adenocarcinoma (PDAC). PDAC are malignant tumors with high-level autophagy [84]. However, cilia are absent in human and mouse PDAC tissues compared to highly ciliated normal tissues [27]. In other word, cilia loss fails to downregulate autophagy in these malignant cells. Although cilia and cilia-dependent Shh have emerged as possible regulators of autophagy, more insightful mechanisms of the regulating system remain to be elucidated.

### Does the crosstalk between cilia and autophagy influence cancer development?

The first link between autophagy and cancer is from the studies of Beclin 1, an essential protein for autophagy initiation [85, 86]. Unlike other tumor suppressors, *Beclin 1* is characterized as a haploid-insufficient tumor suppressor gene, monoallelic mutations of which lead to defective function [85, 86]. Interestingly, only premalignant tumors, but not malignant tumors, are observed in autophagy-deficient mice by knockout of *atg5* or *atg7,* two autophagy essential genes, suggesting a suppressive role of autophagy in cell transformation [87]. In contrast to the function of autophagy in transformation, high level of autophagy is required in malignant tumors, including PDAC and non-small cell lung cancer, to maintain the high level of metabolism [84, 88, 89]. These data suggest a dual role of autophagy in cancer development (Fig. 3). One possible explanation is that autophagy plays different roles in cell transformation and transformed cell progression. At the early stage of cancer development, autophagy can degrade harmful factors, including aggregated proteins and aged mitochondria, to prevent cells from accumulating genomic mutations [89–92]. After cancer cell transformation, autophagy can provide substrates for high-level metabolism and prevent toxic product accumulation, both of which promote cancer survival and proliferation [89, 92]. Additionally, autophagy may also have function inside tumor stroma via the altered secretory products and surface characters [93–98]. Emerging studies suggested that autophagy contributes to starvation and hypoxia-evoked angiogenesis, which promotes tumor stroma accessing more nutrients [99–101]. Activated autophagy in cancer-associated fibroblasts provides more metabolic products to ‘feed’ high proliferative cancer cells with enhanced energy demands [102–105]. Regarding immunity changes in tumor stroma are important for cancer development, studies to demonstrate whether and how autophagy affects immunosurveillance will give more insightful information of autophagy and tumorigenesis [93].

As discussed above, cilia can restrict mitosis and inhibit abnormal cell proliferation [7, 17, 22]. In this way, theoretically, cilia serve as negative regulator of cancer development by providing additional checkpoint of cell cycle progression.

In ciliated cells, the presence of cilia might positively regulate autophagy, preventing metabolic waste accumulation and constitutive cellular damage, which is a potent factor in inducing cancer development [74, 92]. However, in malignant cells, why the loss of cilia and upregulated autophagy are associated together is still poorly understood. Reversely, autophagy has dual roles in ciliogenesis by degrading essential or suppressive cilia-related proteins. If autophagy plays a role in cancer through cilia, how cells modulate the switch to turn on/off cilia expression will be an important point to be addressed. Although the interplay between cilia and autophagy has emerged, recent studies may have just begun to touch a small tip of a giant iceberg. Future studies will hopefully provide more evidences to reveal the complicated connections between cilia and autophagy.

## Conclusion

The list of cilia’s functions is growing fast. As discussed above, cilia can restrict mitosis and inhibit abnormal cell proliferation [7, 17, 22]. In this way, theoretically, cilia serve as negative regulator of cancer development by providing additional checkpoint of cell cycle progression. Interestingly, the bidirectional interplay between cilia and autophagy is emerging as a new field for future studies. Autophagy selectively turns on/off cilia formation by alternatively degrading ciliary essential protein, IFT20, or suppressive protein, OFD1. The mechanisms modulating this switch are still unknown. Involved in autophagy initiation, cilia enhance autophagy flux through cilia-related Shh. Whether and how other cilia-related signaling(s) participate(s) autophagy regulation remains unclear. Given the broad functions of cilia and autophagy in the regulation of cell proliferation and metabolism, discovery of drugs specifically targeting these two regulators will provide a wide therapeutic approach for cancer and other diseases.

## Abbreviations

Shh: Sonic Hedgehog signaling
IFT: intraflagellar transport
OFD1: oral-facial-digital syndrome 1
PCP: planar cell polarity
PDAC: pancreatic ductal adenocarcinoma
